# Supplementary material for: Wide-spread brain activation and reduced CSF flow during avian REM sleep
Source: Nat Commun. 2023 Jun 5;14:3259. doi: 10.1038/s41467-023-38669-1 (PMC10241905; doi:10.1038/s41467-023-38669-1)
Supplement: Supplementary file 1 — Supplementary Information [file 41467_2023_38669_MOESM1_ESM.pdf]

Supplementary Information for

Wide-spread brain activation and reduced CSF flow during avian REM sleep

Gianina Ungurean†*, Mehdi Behroozi†*, Leonard Böger, Xavier Helluy, Paul-Antoine Libourel, Onur Güntürkün, and Niels C. Rattenborg
Correspondence to: mehdi.behroozi@ruhr-uni-bochum.de & gianina.ungurean@bi.mpg.de

Number of Supplementary Figures: 10
Number of Supplementary Tables: 2

Supplementary Figures

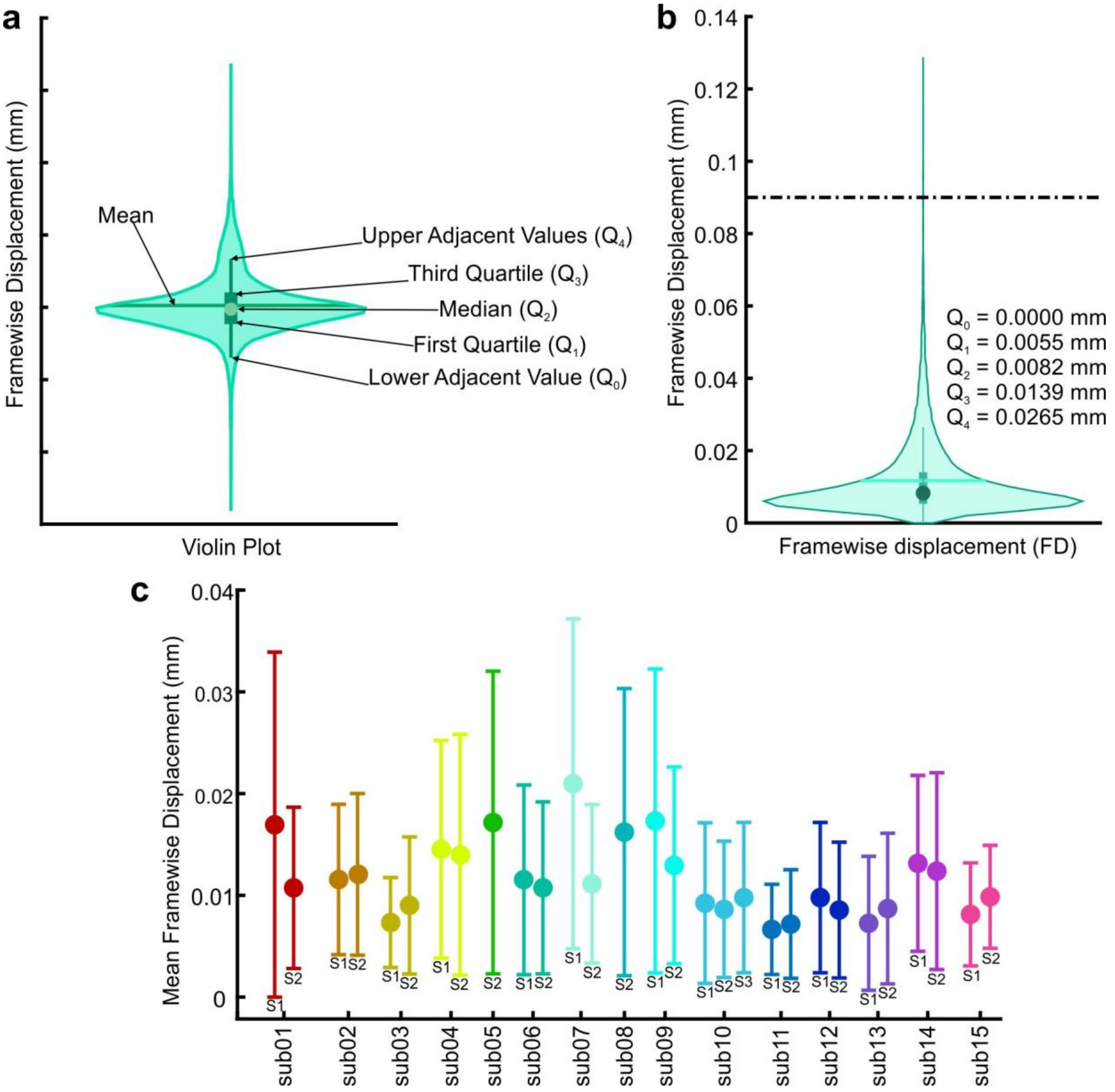

Supplementary Figure 1. Quality control measures.

a how to read a violin plot. **b** Distribution of the framewise displacement (FD) over all subjects (n = 29 sessions over 15 pigeons). FD quantifies the movement of the head from one volume to the next one. The FD values were higher than 0.09 (~20% of voxel size, the threshold used in this study) on only 25 volumes over 29 fMRI sessions. **c** Mean FD of fifteen single subjects sub01 to sub15. Error bars indicate FD standard deviation (SD) over a single fMRI session. Session 1 (S1); Session 2 (S2).

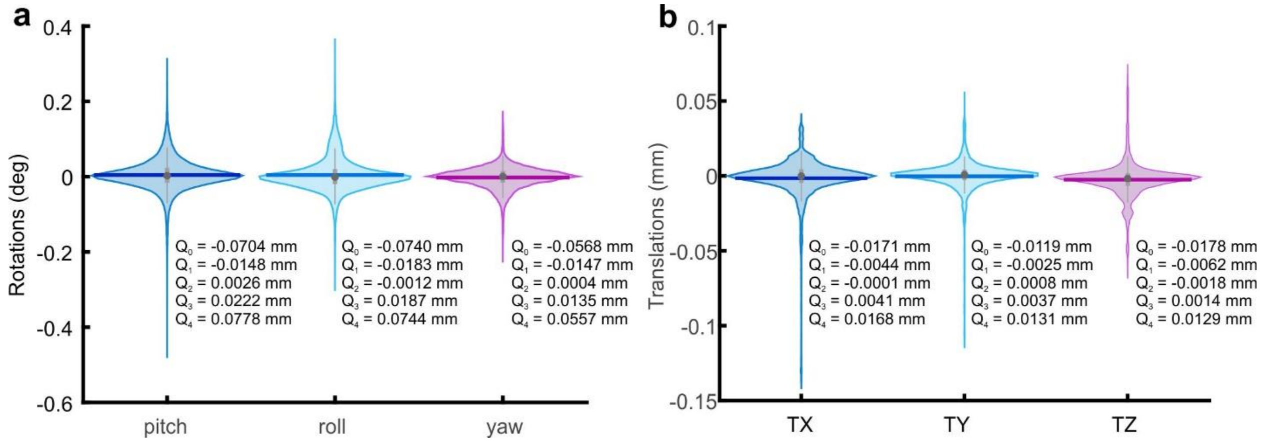

Supplementary Figure 2. Characterization of the head movements.

Distribution of estimated motion parameters over all subjects (n = 29 sessions over 15 pigeons). **a** Estimated head rotations. **b** Estimated translations. Motion parameters were estimated by a 3D rigid body model with six degrees of freedom for translation (x, y, and z-direction) and rotation (pitch, roll, and yaw). Only 0.02% of volumes had estimated translations higher than 0.05 mm (over 29 fMRI sessions). Check Supplementary Figure 1a to read a violin plot.

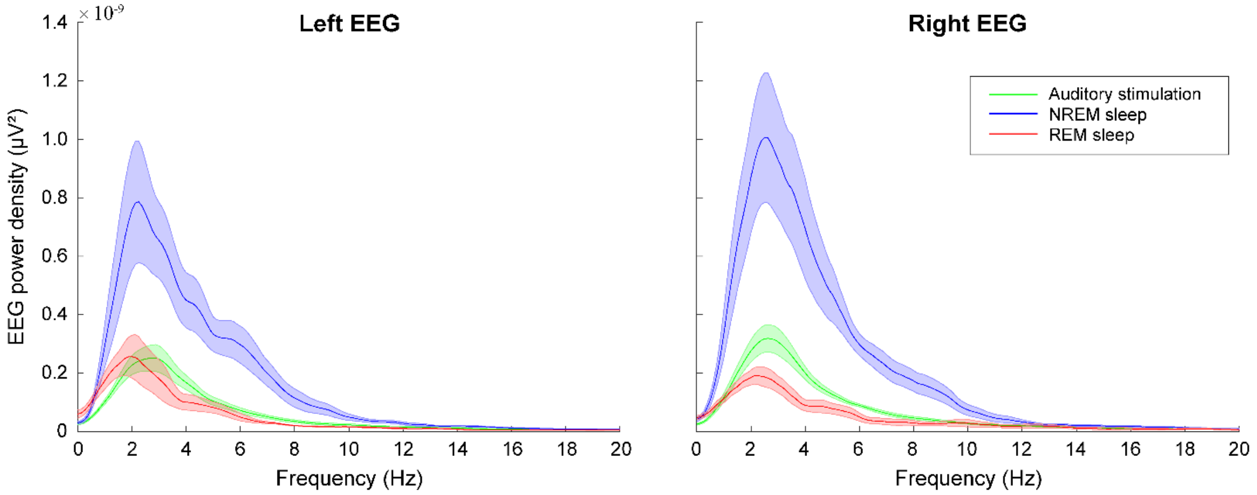

Supplementary Figure 3. Activity in left and right hyperpallial EEG corresponding to behaviorally defined sleep states and auditory stimulation.

Power spectral density (PSD) was calculated for bouts scored as NREM (blue) or REM (red) sleep using the same behavioral criteria as for sleep scoring inside the fMRI scanner (n =6 pigeons). PSD occurring during auditory stimulation is shown in green. Data presented as mean ± SEM of the average curve per individual from n=6 birds.

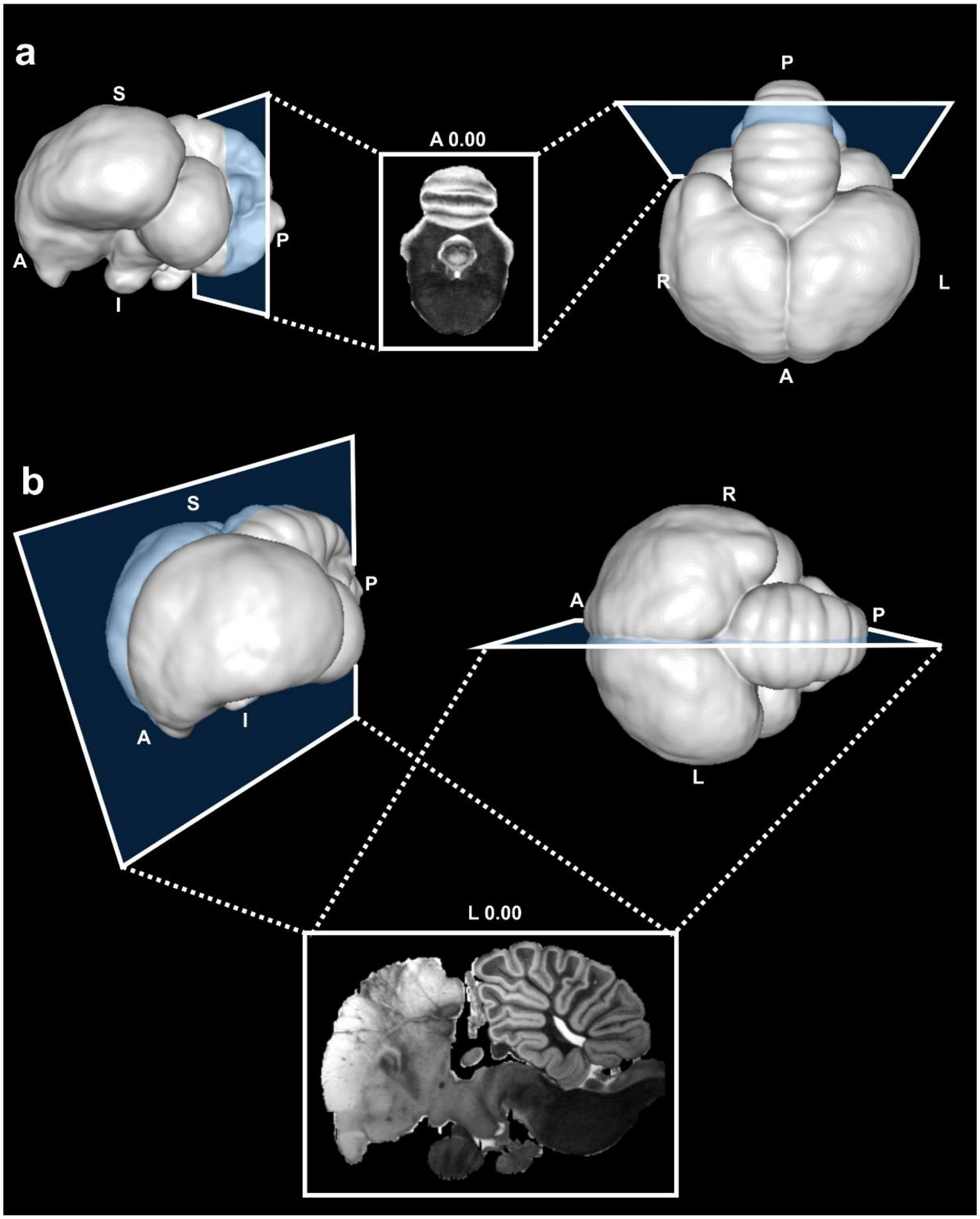

Supplementary Figure 4. Definition of a coordinate system of reference for a Budapest pigeon brain using the high-resolution anatomical image.

Because the brain anatomy of the Budapest pigeon is slightly different from that of other varieties of *Columba livia* commonly used in research, it is necessary to define a breed-specific coordinate system of reference based on Budapest pigeon-specific landmarks. **a** Frontal section showing the anterior A 0.00 plane of the brain. **b** Lateral section. L 0.00 defines the mid-sagittal plane of the brain.

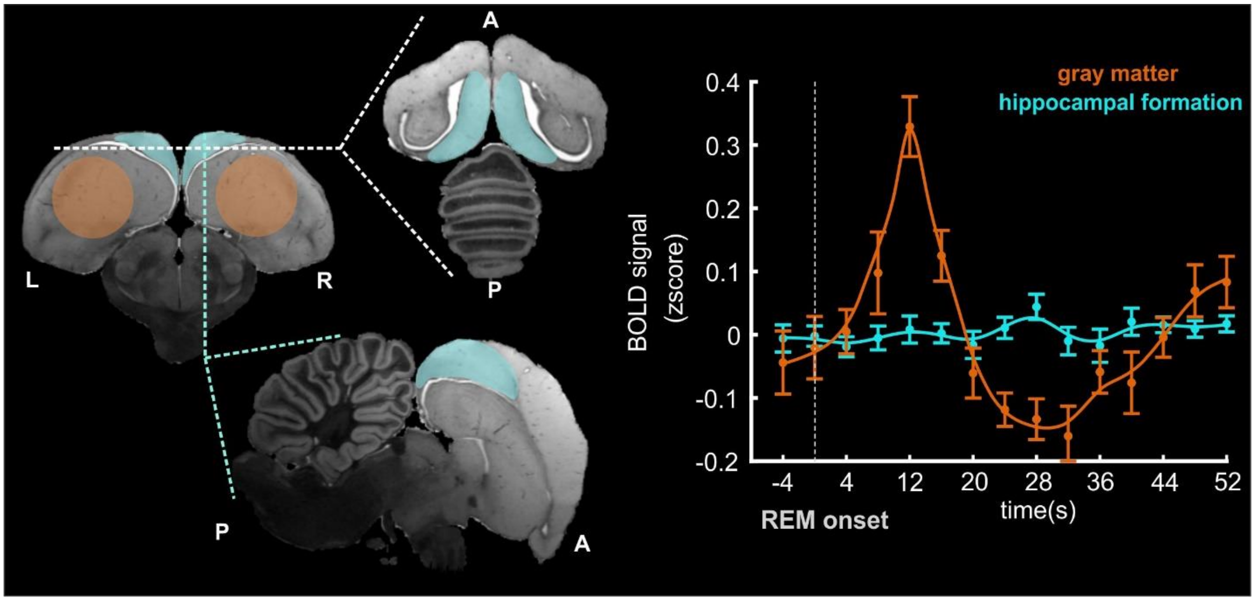

Supplementary Figure 5. The hippocampal formation signal during REM sleep.

The left panel shows the position of the pigeon hippocampal formation and the selected gray matter, previously shown to have increased BOLD signal during REM sleep, in three different views, axial, horizontal, and sagittal. The BOLD signal was extracted from voxels in the grey matter (red mask) and hippocampal formation (green mask). When compared to NREM sleep, the BOLD signal increased in the grey matter, but not the hippocampal formation, during REM sleep. Error bars reflect the standard error of the mean across subjects.

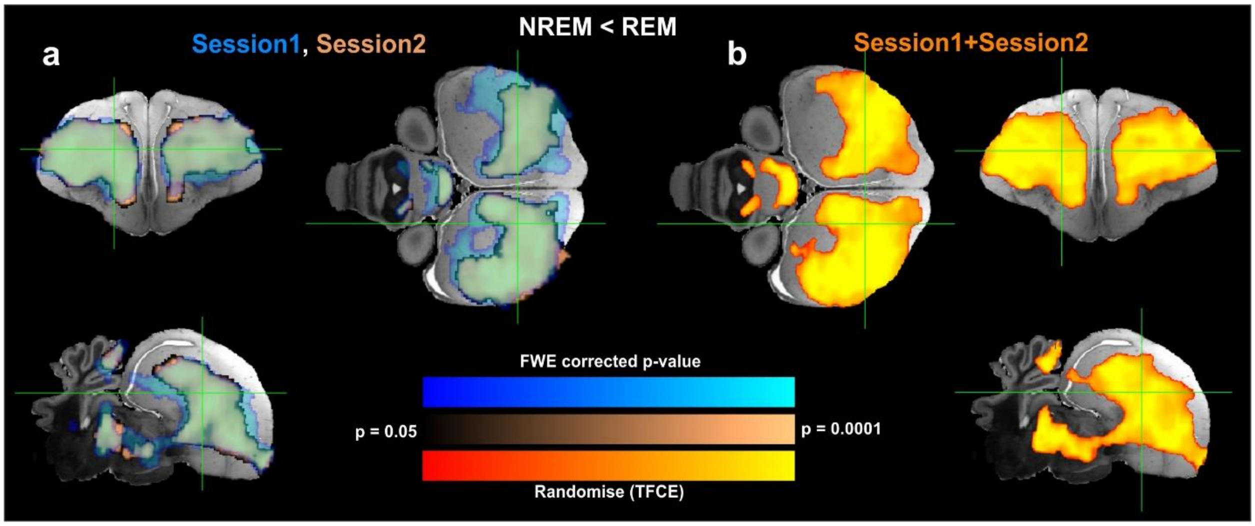

Supplementary Figure 6. Repeatability of REM sleep results.

a Activation patterns of REM > NREM from session 1 (blue, *n* = 14) and session 2 (copper, *n* = 14). The activation patterns are overall very similar (green), indicating that the results are reproducible. A repeated measures ANOVA (session 1 and 2 as fixed factor and subjects as a random factor) demonstrated no difference between both sessions (one sided paired t-test randomize, established in FSL). **b** The activation patterns of REM > NREM after averaging data from two independent sessions.

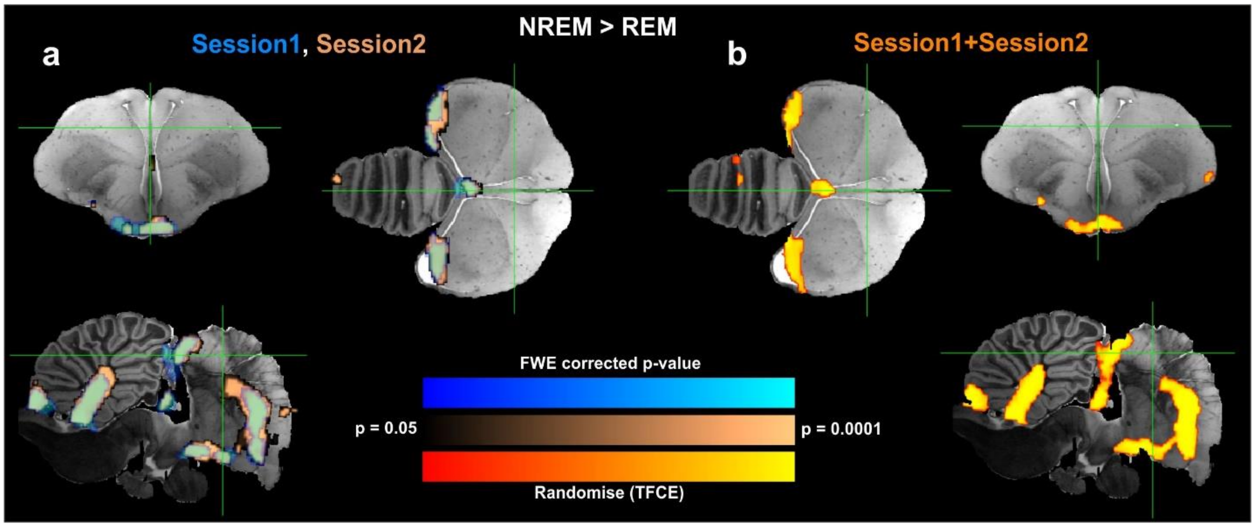

Supplementary Figure 7. Repeatability of NREM sleep results.

a Activation patterns of NREM > REM from session 1 (blue, n = 14) and session 2 (copper, n = 14). The activation patterns are overall very similar, indicating that the results are reproducible. A repeated measures ANOVA (session 1 and 2 as fixed factor and subjects as a random factor) demonstrated no difference between both sessions (one sided paired t-test randomize, established in FSL). **b** The activation patterns of NREM > REM after averaging data from two independent sessions.

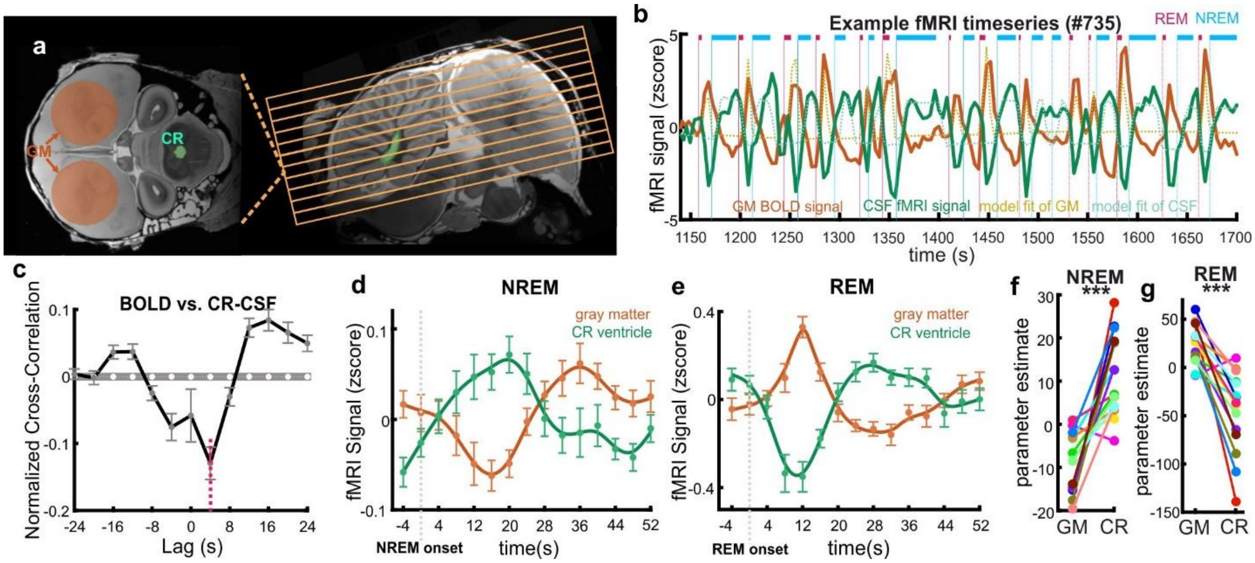

Supplementary Figure 8. Gray matter BOLD and CSF signal correlations are reproducible from the CR of the IV ventricle.

a Position of acquired functional slices and selected ROIs relative to the high-resolution anatomical image. Eleven slices have been positioned in a way to cover the whole telencephalon and ventricular system of the pigeon brain (yellow slices). The BOLD signal was extracted from the voxels in the pigeon's telencephalon (the red mask, a sphere with a diameter of 6.5 mm centered at the entopallium to cover most of the telencephalon). The CSF signal was averaged from the CR (green mask). The CR voxels appear much brighter than the surrounding areas in the high-resolution anatomical image. **b** Example time-series of the telencephalic BOLD and CSF signals depicting changes in both signals relative to bouts of NREM and REM sleep (from a representative pigeon). During sleep, the BOLD signal dynamics are anti-correlated to the CSF signal. The BOLD signal increases during REM sleep when CSF flow decreases. The CSF flow increases during NREM sleep when the BOLD signal decreases. **c** Mean cross-correlation between BOLD and CSF signals (n = 29, 15 pigeons). The BOLD signal is significantly anti-correlated with the CSF signal. The gray shaded region shows the 95% confidence interval of shuffled data. The white dashed line in the shaded region represents the mean correlation from shuffled data at each time lag. Error bars denote the standard error of the mean (SEM) across different lags. The cross-correlation at +4 s lag demonstrates the strongest coupling between BOLD and CSF signal (-0.13, p = 2e-04 two-side permutation test, red dashed line). **d,e** Average time courses in GM-BOLD and CSF signals locked on NREM and REM sleep onset, respectively (n = 29 sessions,15 pigeons). Error bars represent SEM. **f,g** Parameter estimates during NREM and REM sleep, respectively (n = 15). Each colored circle shows the average beta estimate over the GM mask and CR mask for each bird. CSF inflow increases compared to the GM-BOLD signal during NREM sleep (t(14) = 6.3, p = 7e-05, two-tailed paired-sample t-test), and the opposite occurs during REM sleep (t(14) = -5.7, p = 1e-04, two-tailed paired-sample t-test).

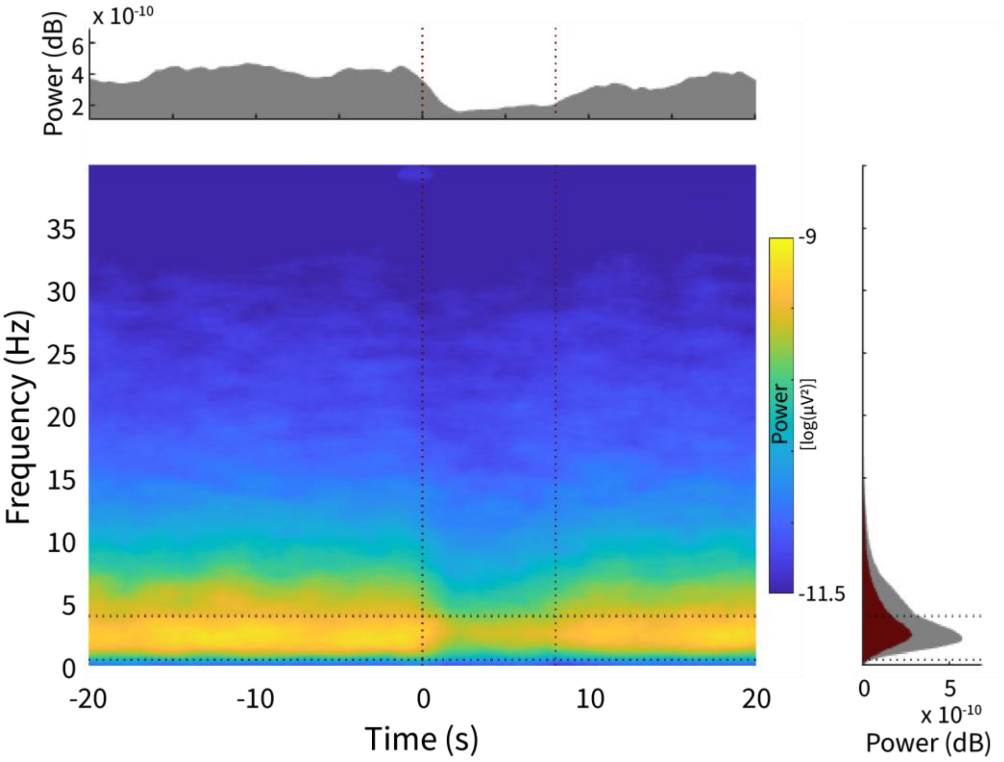

Supplementary Figure 9. Birds show awake brain activity during auditory stimulation.

Main panel: Time-frequency representation of the mean EEG (power spectral density) response upon auditory stimulation. Vertical dashed lines represent the stimulation window. Horizontal dashed lines show the delta power band (0.5-4 Hz). Top panel: average time course of the delta power band during stimulation. Right panel: average power spectra prior to (gray) and during stimulation (dark red).

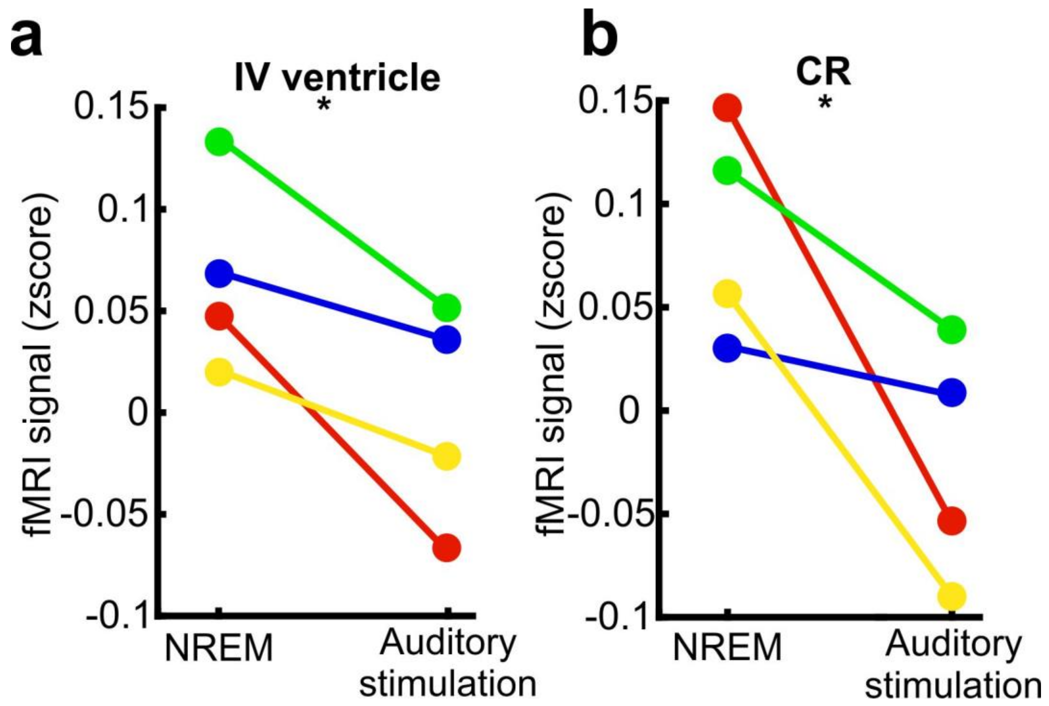

Supplementary Figure 10. Pigeons showed reduced CSF flow during auditory stimulation compared to NREM sleep.

We compared the fMRI signals from the IV ventricle excluding the cerebellar recess (CR), and the CR itself, during NREM sleep and auditory stimulation. A single fMRI mean image was created for each auditory stimulation and NREM sleep condition by taking the mean of the two volumes 4 s after the condition onset (to account for hemodynamic response delay). **a** CSF inflow in IV was significantly reduced during auditory stimulation ($t(3) = 4.02$, $p = 0.009$, two-tailed paired sample t-test). **b** The results for the CR were similar to IV ($t(3) = 4.5$, $p = 0.008$, two-tailed paired sample t-test).

Supplementary Tables

Supplementary Table 1. Alphabetized list of abbreviations

Abb.	ROI name	Abb.	ROI name
**AC**	n. Accumbens	**L3**	Field-L3
**AD**	Dorsal Arcopallium	**LoC**	Locus coeruleus
**AI**	Arcopallium intermedium	**M**	Mesopallium
**AM**	Arcopallium mediale	**MC**	Mesopallium caudale
**AP**	Posterior arcopallium	**MFD**	Mesopallium frontodorsale
**AQP4**	Aquaporin 4	**MFV**	Mesopallium frontoventrale
**AV**	Arcopallium ventrale	**MIVm**	Mesopallium intermedioventralle, pars medialis
**BOLD**	Blood-Oxygen-Level-Dependent	**MIVl**	Mesopallium intermedioventralle, pars lateralis
**CbL**	Lateral cerebellar nucleus	**MLd**	Nucleus mesencephalicus lateralis, pars dorsalis
**CbM**	Medial cerebellar nucleus	**MLv**	Nucleus mesencephalicus lateralis, pars ventralis
**CDL**	Area Corticoidea dorsolateralis	**NCC**	Nidopallium caudocentrale
**CR**	Cerebellar recess of the fourth ventricle	**NCL**	Nidopallium caudolaterale
**CSF**	Cerebrospinal fluid	**NCM**	Nidopallium caudomediale
**DLA**	Nucleus dorsolateralis anterior thalami	**NFL**	Nidopallium frontolaterale
**DMA**	Nucleus dorsomedialis anterior thalami	**NFT**	Nidopallium frontotrigeminale
**E**	Entopallium	**NIL**	Nidopallium intermediolaterateralis
**EEG**	Electroencephalogram	**NIMl**	Nidopallium intermediolaterale
**fMRI**	functional Magnetic Resonance Imaging	**NMm**	Nidopallium intermediomediale
**FRM**	Formatio reticularis medialis mesencephali	**NREM**	Non-rapid eye movement
**GP**	globus pallidus	**NSTL**	Bed nucleus of the stria terminalis
**HA**	Hyperpallium Apicale	**OV**	n. ovoidalis
**HD**	Hyperpallium Densocellulare	**PCV**	Cerebellovestibular process
**HI**	Hyperpallium Intercalatum	**PV**	Nucleus posteroventralis thalami
**Hp**	Hippocampal formation	**REM**	Rapid eye movement
**Hy**	Hypothalamus	**RARE**	Rapid Acquisition with Relaxation Enhancement
**ICo**	Nucleus intercollicularis	**Rt**	n. Rotundus
**IHA**	Interstitial n. of Hyperpallium Apicale	**SCE**	Stratum cellulare externum
**III**	3rd ventricle	**SLu**	n. semilunaris
**Imc**	n. isthmi pars magnocellularis	**StL**	Lateral striatum
**Ipc**	n. isthmi pars parvocellularis	**StM**	Medial striatum
**ITI**	Inter trial interval	**SVN**	Dorsal part of the superior vestibular nucleus
**IV**	4th ventricle	**TPO**	Area temporo-parieto-occipitalis
**LHy**	Lateral Hypothalamus	**VL**	Lateral ventricle
**L1**	Field-L1	**VP**	Ventral pallidum
**L2**	Field-L2		

Supplementary Table 2. List of subjects involved in the different analysis.

Animal Identity	Sex (male / female)	REM and NREM (n = 15)	Left eye open (n = 11)	Right eye open (n = 12)	Both eyes open (n = 9)	Auditory stimulation (n = 4)	EEG and auditory stimulation out of scanner (n = 6)
**29**	m	✓	✓	✓	✓	x	x
**33**	f	✓	✓	✓	✓	✓	x
**34**	f	✓	✓	✓	✓	x	✓
**35**	f	✓	x	✓	✓	x	x
**36**	m	✓	✓	✓	✓	✓	x
**37**	f	✓	✓	✓	✓	x	x
**38**	f	✓	✓	✓	✓	✓	x
**52**	m	✓	✓	x	x	x	x
**69**	f	✓	✓	✓	x	✓	x
**78**	m	✓	✓	✓	✓	x	✓
**84**	m	✓	✓	✓	✓	x	✓
**712**	m	✓	x	✓	x	x	✓
**721**	m	✓	x	x	x	x	✓
**735**	f	✓	✓	✓	x	x	x
**736**	m	✓	x	x	x	x	✓
