# Supplementary material for: Wide-spread brain activation and reduced CSF flow during avian REM sleep
Source: Nat Commun. 2023 Jun 5;14:3259. doi: 10.1038/s41467-023-38669-1 (PMC10241905; doi:10.1038/s41467-023-38669-1)
Supplement: Supplementary file 3 — Description of Additional Supplementary Files Document [file 41467_2023_38669_MOESM3_ESM.pdf]

Description of Additional Supplementary Files Document

Supplementary Movie Legend

Supplementary Movie 1: Sample video of a Budapest pigeon cycling between NREM and REM sleep during an fMRI recording. The bird is head-fixed inside the fMRI machine and two mirrors are placed on each side of the head to visualize the state of the eyes, pupils, and beak. Note that the birds' pupils are visible through the closed eyelids. The video starts with the bird in NREM sleep, as indicated by closure of the eyes, dilated pupils, and no eye or bill movements. The bird then enters REM sleep, characterized by eye closure, rapidly constricting pupils, and rapid eye and bill movements. The bird then returns to NREM sleep.
