# Supplementary material for: Wide-spread brain activation and reduced CSF flow during avian REM sleep
Source: Nat Commun. 2023 Jun 5;14:3259. doi: 10.1038/s41467-023-38669-1 (PMC10241905; doi:10.1038/s41467-023-38669-1)
Supplement: Supplementary file 5 — Reporting Summary [file 41467_2023_38669_MOESM5_ESM.pdf]

nature portfolio

Corresponding author(s): Mehdi Behroozi, Gianina Ungurean1

Last updated by author(s): Apr 7, 2023



# Reporting Summary

Nature Portfolio wishes to improve the reproducibility of the work that we publish. This form provides structure for consistency and transparency in reporting. For further information on Nature Portfolio policies, see our Editorial Policies and the Editorial Policy Checklist.

## Statistics

For all statistical analyses, confirm that the following items are present in the figure legend, table legend, main text, or Methods section.

| n/a | Confirmed | |
|---|---|---|
| ☐ | ☒ | The exact sample size (*n*) for each experimental group/condition, given as a discrete number and unit of measurement |
| ☐ | ☒ | A statement on whether measurements were taken from distinct samples or whether the same sample was measured repeatedly |
| ☐ | ☒ | The statistical test(s) used AND whether they are one- or two-sided<br>*Only common tests should be described solely by name; describe more complex techniques in the Methods section.* |
| ☐ | ☒ | A description of all covariates tested |
| ☐ | ☒ | A description of any assumptions or corrections, such as tests of normality and adjustment for multiple comparisons |
| ☐ | ☒ | A full description of the statistical parameters including central tendency (e.g. means) or other basic estimates (e.g. regression coefficient) AND variation (e.g. standard deviation) or associated estimates of uncertainty (e.g. confidence intervals) |
| ☐ | ☒ | For null hypothesis testing, the test statistic (e.g. *F*, *t*, *r*) with confidence intervals, effect sizes, degrees of freedom and *P* value noted<br>*Give P values as exact values whenever suitable.* |
| ☒ | ☐ | For Bayesian analysis, information on the choice of priors and Markov chain Monte Carlo settings |
| ☒ | ☐ | For hierarchical and complex designs, identification of the appropriate level for tests and full reporting of outcomes |
| ☒ | ☐ | Estimates of effect sizes (e.g. Cohen's *d*, Pearson's *r*), indicating how they were calculated |

*Our web collection on statistics for biologists contains articles on many of the points above.*

## Software and code

Policy information about availability of computer code

| | |
|---|---|
| Data collection | We used custom MATLAB (2020b, the Mathwork, Natick, MA USA) to recode videos, present auditory stimuli, recode EEG signal, and Paravision 6.0 to measure fMRI data. |
| Data analysis | Video analyzes were performed using custom-written MATLAB (2020b) to score REM, NREM , and the open-eye condition.<br>EEG signals were analyzed in MATLAB (2020b).<br><br>All processing of fMRI data was performed using tools from the FMRIB Software Library (https://fsl.fmrib.ox.ac.uk/fsl/fslwiki/FSL, version 5.0.9), Analysis of Functional NeuroImages (AFNI, version 20.0.09 https://afni.nimh.nih.gov/), and Advanced Normalization Tools (ANTs, http://stnava.github.io/ANTs/, version 2.1 1) software.<br><br>Custome code for fMRI analysis: https://doi.org/10.5281/zenodo.7801994<br><br>Custome code for EEG analysis: https://doi.org/10.17617/3.JFRZHS<br><br>All linear mixed model analyses were performed in R (version 4.2.1) using the lmer package, version 1.1-32.<br><br>The 3D MRI images were visualized with the Mango software (http://ric.uthscsa.edu/mango/, version 4.1). |

For manuscripts utilizing custom algorithms or software that are central to the research but not yet described in published literature, software must be made available to editors and reviewers. We strongly encourage code deposition in a community repository (e.g. GitHub). See the Nature Portfolio guidelines for submitting code & software for further information.

## Data

Policy information about availability of data

All manuscripts must include a data availability statement. This statement should provide the following information, where applicable:

- Accession codes, unique identifiers, or web links for publicly available datasets
- A description of any restrictions on data availability
- For clinical datasets or third party data, please ensure that the statement adheres to our policy

The fMRI data generated in this study are publicly available in BIDS format and have been deposited in OpenNeuro (Accession number: ds004465) as functional MRI of sleeping pigeon (https://openneuro.org/datasets/ds004465).

The EEG data are available at https://doi.org/10.17617/3.JFRZHS .
All information regarding the timing of REM and NREM, obtained from videos, has already been shared within the fMRI data.

## Human research participants

Policy information about studies involving human research participants and Sex and Gender in Research.

| | |
|---|---|
| Reporting on sex and gender | not applicable |
| Population characteristics | not applicable |
| Recruitment | not applicable |
| Ethics oversight | not applicable |

Note that full information on the approval of the study protocol must also be provided in the manuscript.

# Field-specific reporting

Please select the one below that is the best fit for your research. If you are not sure, read the appropriate sections before making your selection.

☒ Life sciences ☐ Behavioural & social sciences ☐ Ecological, evolutionary & environmental sciences

For a reference copy of the document with all sections, see nature.com/documents/nr-reporting-summary-flat.pdf

# Life sciences study design

All studies must disclose on these points even when the disclosure is negative.

**Sample size**

No sample-size calculation was perfromed.
This sample size in our study (15 pigeons) have shown to be sufficient to investigate avian brain (Behroozi et al., 2020) and rodent brain (Liu et al., 2019) using fMRI. In addtion, this sample size is in line with comparable orientation studies in sleep stages in rodent (e.g. 7 rats in Begrel A., Tanter M., et al., Nat Commun, 2018).

References:
Behroozi, M., Helluy, X., Ströckens, F. et al. Event-related functional MRI of awake behaving pigeons at 7T. Nat Commun 11, 4715 (2020). https://doi.org/10.1038/s41467-020-18437-1

Liu, C., Yen, C.C., Szczupak, D. et al. Anatomical and functional investigation of the marmoset default mode network. Nat Commun 10, 1975 (2019). https://doi.org/10.1038/s41467-019-09813-7

Bergel, A., Deffieux, T., Demené, C. et al. Local hippocampal fast gamma rhythms precede brain-wide hyperemic patterns during spontaneous rodent REM sleep. Nat Commun 9, 5364 (2018). https://doi.org/10.1038/s41467-018-07752-3

**Data exclusions**

Due to low amounts of NREM and REM sleep. the second session of one pigeon was excluded from the analyses.

**Replication**

To check the reproducibility and stability of the results, rsfMRI data of all animals were recorded twice on different days. The activation patterns are overall very similar and indicate reproducible results. A repeated measures ANOVA (day 1 and 2 as fixed factor and subjects as a random factor) demonstrated no difference between both days (paired t-test randomize, established in FSL).

**Randomization**

For the current study, 15 pigeons were randomly selected from local breeders. Six of them were randomly chosen to have EEG electrodes implanted. For the auditory experiment, four animals were randomly selected to receive auditory stimuli to wake them up while inside the scanner.

| | |
|---|---|
| Blinding | Blind analysis was not required since we were not interested in differences in group performance. |

# Reporting for specific materials, systems and methods

We require information from authors about some types of materials, experimental systems and methods used in many studies. Here, indicate whether each material, system or method listed is relevant to your study. If you are not sure if a list item applies to your research, read the appropriate section before selecting a response.

## Materials & experimental systems

| n/a | Involved in the study |
|---|---|
| ☒ | ☐ Antibodies |
| ☒ | ☐ Eukaryotic cell lines |
| ☒ | ☐ Palaeontology and archaeology |
| ☐ | ☒ Animals and other organisms |
| ☒ | ☐ Clinical data |
| ☒ | ☐ Dual use research of concern |

## Methods

| n/a | Involved in the study |
|---|---|
| ☒ | ☐ ChIP-seq |
| ☒ | ☐ Flow cytometry |
| ☐ | ☒ MRI-based neuroimaging |

# Animals and other research organisms

Policy information about studies involving animals; ARRIVE guidelines recommended for reporting animal research, and Sex and Gender in Research

| | |
|---|---|
| Laboratory animals | Fifteen adult domestic pigeons (Columba livia, Budapest highflyer variety; 7 females and 8 males, 2-3 years old, genetically sexed), obtained from a local breeder, were used in this study. |
| Wild animals | The study did not involve wild animals. |
| Reporting on sex | We used both sexes in our study. The sex-based analyses have already been included. The results indicated no sex effect on our results and the data for both sexes were combined in main text. |
| Field-collected samples | No field collected samples were used in the study. |
| Ethics oversight | Approved by the Landesamt für Natur, Umwelt und Verbraucherschutz Nordrhein-Westfalen (LANUV),<br>Application number: Az.: 81-02.04.2021.A240 |

Note that full information on the approval of the study protocol must also be provided in the manuscript.

# Magnetic resonance imaging

## Experimental design

| | |
|---|---|
| Design type | resting-state |
| Design specifications | Out of the 15 pigeons, 14 had two recording sessions of resting-state, and one had only one. The recording durations were between 60 to 90 min. A subgroup of six birds, were recorded outside the scanner during the habituation phase using video and EEG measurements. The recordings lasted between 60 and 90 minutes. |
| Behavioral performance measures | The sleep states were manually scored using the video recordings. To facillitate the scoring process, actimetry signals were calculated for the eyes, irises, and bill region and visualized together with the video recordings. NREM sleep was characterized by bilateral eye closure, stable breathing (as visible in the videos and bill actimetry signals), immobility of the eyes, and absence of bill movements other than those related to breathing. REM sleep was characterized by bilateral eye closure with movements of the eyes, bill, and/or the collapse of head feathers held erect during preceding NREM sleep. As described previously (Ungurean et al., 2021), rapid constrictions and dilations of the iris are closely associated with REM sleep. Thus, rapid iris movements were also used to identify bouts of REM sleep and their absence to confirm NREM sleep. |

## Acquisition

| | |
|---|---|
| Imaging type(s) | functional and structural scans |
| Field strength | 7T |
| Sequence & imaging parameters | Resting-State fMRI (rs-fMRI) data were acquired using a single-shot multi-slice RARE sequence adapted from Behroozi et al. (2020), with the following parameters: TR=4000ms, TEeff=41.58ms, partial Fourier transform accelerator=1.53, encoding matrix=64×42, acquisition matrix=64×64, FOV=30×30mm2, in-plane spatial resolution=0.47×0.47mm2, |

radio-frequency pulse flip angles for excitation and refocusing=90°/180°, slice thickness=1mm, no slice distance, slice order=interleaved, excitation and refocusing pulse form=scanner vendor gauss512, receiver bandwidth=50,000Hz. To saturate the signals from the eyes to avoid brain image corruption due to eye movements, two saturation slices were positioned manually over the eyes. Each run of the rsfMRI recordings included 1000 to 1450 volumes. To check the reproducibility and stability of the results, rsfMRI data of all animals were recorded twice on different days.
High-resolution T2-weighted anatomical images were acquired using a RARE sequence for better spatial normalization. Scan parameters were as follows: TR=2000 ms, TEeff=50.72ms, RARE factor=16, number of averages=1, FOV=25×25×15mm3, matrix size=128×128×64, spatial resolution=0.2×0.2×0.23mm3. The total scanning time was 17min.

Area of acquisition: whole brain

Diffusion MRI: ☐ Used ☒ Not used

## Preprocessing

Preprocessing software: All fMRI data processing was performed using tools from the FMRIB Software Library (https://fsl.fmrib.ox.ac.uk/fsl/fslwiki/FSL, version 5.0.9), the Analysis of Functional NeuroImages (AFNI, version 20.0.09 https://afni.nimh.nih.gov/), and Advanced Normalization Tools (ANTs, http://stnava.github.io/ANTs/) software.

Normalization: We co-registered each rs-fMRI data to the corresponding T2-weighted anatomical images using affine linear registration (12 degrees of freedom). A population-based template was generated using antsMultivariateTemplateConstruction.sh script. After analyzing individual subjects, the results were normalized to the population-based template using FMRIB's Nonlinear Image Registration Tool (FNIRT) for group analysis.

Normalization template: A population-based template was generated using antsMultivariateTemplateConstruction.sh script. For visualizing the results, the group results were non-linearly warped to the high-resolution post-mortem anatomical image. 3D MRI images were visualized using the MANGO software (http://ric.uthscsa.edu/mango/, version 4.1).

Noise and artifact removal: Six estimated motion parameters were estimates using MCFLIRT function of FSL which modeled in all GLMs as nuisance regressors to account for any residual effects of animal movement.

Volume censoring: The censoring of high motion frames was applied to estimate the amount of head movement within each resting-state time series. The threshold for framewise displacement (i.e., volume to volume movement) was set as 0.9 mm (less than 20% of voxel size). The results indicated that only 25 volumes of all 29 rs-fMRI sessions had an FD-value higher than 0.9 mm (after upscaling voxel size by a factor of 10).

## Statistical modeling & inference

Model type and settings: Mass univariate; fixed-effects within subject to combine fMRI data across scan runs; random-effects across subjects for second-level analyses

Effect(s) tested: The activation patterns are overall very similar and indicate reproducible results. A repeated measures ANOVA (day 1 and 2 as fixed factor and subjects as a random factor) demonstrated no difference between both days (paired t-test randomize, established in FSL).

Specify type of analysis: ☒ Whole brain ☐ ROI-based ☐ Both

Statistic type for inference (See Eklund et al. 2016): For the REM vs. NREM group analysis, contrasts of interest from second-level analyses were then taken into the higher-level analysis using the nonparametric FSL's Randomise function. Statistical significance was assessed using permutation testing with 5,000 permutations, Threshold-Free Cluster Enhancement (TFCE), and a family-wise error (FWE) corrected cluster p-value of p = 0.05. For the open eye condition group analysis, contrasts of interest from second-level analyses were then taken to the higher-level analysis using the mixed-effect model (FLAME1). Higher-level results were thresholded using activation levels determined by Z>2.3 (p<0.01) and FWE cluster significance threshold of p=0.05.

Correction: family-wise error (FWE)

## Models & analysis

| n/a | Involved in the study |
|---|---|
| ☒ | ☐ Functional and/or effective connectivity |
| ☒ | ☐ Graph analysis |
| ☒ | ☐ Multivariate modeling or predictive analysis |
